# Nanomaterial-mediated modulation of gut microbiota for precision diagnosis and treatment of colorectal cancer: a comprehensive review

**DOI:** 10.3389/fphar.2026.1758617

**Published:** 2026-02-24

**Authors:** Ying Yang, Shihan Feng, Yunqing Cheng, Min Zhang

**Affiliations:** College of Life Sciences, Jilin Normal University, Siping, Jilin, China

**Keywords:** colorectal cancer, diagnosis and therapy, drug resistance, gut microbiota, nanomaterials

## Abstract

The critical role of gut microbiota in the initiation and progression of colorectal cancer (CRC) has garnered widespread recognition. Leveraging their precise targeting capabilities and programmable properties, nanomaterials are emerging as novel strategy for modulating the interplay between microbiota and tumors. This comprehensive review examines functional nanomaterials—including nanoparticles, nanocapsules, and nanoreactors—and elucidates their mechanisms of action in remodeling the CRC immune microenvironment and potentiating responses to chemoradiotherapy and immunotherapy through microbiota modulation. We further highlight the synergistic value of nanomaterials in multimodal CRC theranostics: (i) serving as microbiota modulation carriers for integrated diagnosis and therapy; (ii) activating systemic anti-tumor immunity via the gut-microbiota-immune axis; and (iii) targeting CRC drug resistance and metastasis. Finally, we discuss challenges associated with clinical translation, including assessment of long-term nanomaterial biosafety and optimization of personalized microbiota intervention protocols. This review provides theoretical foundations and technical insights for developing precision diagnostic and therapeutic strategies for CRC based on nano-microbiota interactions.

## 1 Introduction

Colorectal cancer (CRC) represents a prevalent malignant tumor, ranking as the third most common cancer globally and the second leading cause of cancer-related mortality (Xi and Xu, 2021). In 2020, approximately 1.91 million new CRC cases and 915,000 deaths were reported worldwide, with projections indicating a sustained increase in CRC patients by 2040. Accelerated urbanization and shifting dietary patterns, coupled with factors such as environmental pollutant exposure, excessive processed food intake, high-sugar and high-fat dietary regimes, and alcohol consumption and smoking, have contributed to continuously rising CRC incidence and a troubling trend toward younger onset (Matsuda et al., 2025). Current clinical management of CRC primarily relies on surgical resection and chemoradiotherapy; however, these approaches face critical bottlenecks including high postoperative recurrence rates, pronounced systemic toxicities, and tumor drug resistance. Emerging research demonstrates that gut microbiota influences CRC pathogenesis by modulating inflammatory responses, generating toxic metabolites, inducing oxidative stress, and facilitating immune evasion (Sánchez-Alcoholado et al., 2020). Moreover, the gut microbiome effectively regulates therapeutic efficacy and attenuates toxicity in cancer chemotherapy and immunotherapy (Chrysostomou et al., 2023). Conventional microbiota-based interventions for CRC include probiotic therapy and fecal microbiota transplantation (FMT) (Sepehr et al., 2024; Damman et al., 2012); however, oral probiotic administration suffers from poor bacterial viability and limited colonization efficacy, while FMT faces core challenges encompassing safety risks, difficulties in donor screening and management, unclear mechanisms of action that restrict precise application, and complex regulatory and ethical concerns.

The emergence of nanotechnology has profoundly reshaped the therapeutic approach to CRC. For example, nano-drug delivery systems address key limitations of conventional agents—such as poor solubility, low tolerability, and suboptimal release kinetics—thereby significantly enhancing their therapeutic potential against CRC. Nanocarriers show a strong ability to improve gut microbiota diversity, selectively interact with intestinal microorganisms, eliminate pathogenic bacteria, suppress procarcinogenic metabolite production, and modulate inflammatory responses. Compared with traditional CRC treatments, the combined use of nanotechnology and gut microbiota modulation offers distinct advantages: these systems not only exhibit intrinsic anticancer activity but also integrate diagnostic and tumor microenvironment-regulating functions, leading to further synergistic improvements in treatment outcomes.

This review provides a comprehensive summary of recent advances in nano-therapeutic systems for CRC. It outlines the applications of nanotechnology in CRC diagnosis and prevention, as well as key progress in nanodelivery platforms for targeted probiotic and drug delivery. Furthermore, it highlights emerging nano-enabled strategies for CRC treatment via gut microbiota modulation, including nano-enhanced microbiota therapy, immunotherapy, anti-inflammatory treatment, and chemoradiotherapy. The review also examines representative examples of how nanotechnology is being used to overcome critical challenges such as therapeutic resistance and tumor metastasis in CRC.

While earlier reviews have discussed the role of gut microbiota in CRC or the application of nanomaterials in oncology, the present work distinguishes itself by providing an integrated and up-to-date of nanotechnology that explicitly bridges diagnostics, prevention, and multi-modal therapy through microbiota modulation. This review systematically organizes these emerging approaches into a coherent framework, emphasizing translational challenges, clinical scalability, and the mechanistic interplay between nanomaterials, microbiota, and host immunity. This review aims to offer new perspectives and conceptual frameworks for designing and implementing future strategies for CRC prevention and treatment.

## 2 Nanotechnology improves diagnostic efficiency and prevention of CRC

Early diagnosis and precise prevention are pivotal for reducing the incidence and mortality of CRC. Current clinical screening methods (such as fecal occult blood tests, colonoscopy), while effective in identifying early lesions, have related limitations such as invasiveness, insufficient sensitivity, and poor compliance (Zhang et al., 2023). The breakthrough application of nanotechnology is providing transformative solutions. By designing highly sensitive nano-probes, targeted contrast agents, and miniaturized sensing platforms, novel diagnostic tools that are non-invasive, low-cost, and enable real-time dynamic monitoring can be created, significantly improving the detection rate of early cancerous changes. Future research can further facilitate the clinical translation of nano-diagnostic technologies, strengthen public awareness of active CRC prevention and control, thereby achieving a paradigm shift from “passive treatment” to “active intervention.”

### 2.1 Diagnosis of CRC

Detection of gut microbiota biomarkers provides a new paradigm for early diagnosis of CRC. Nanomaterials have become a focus of research due to their unique surface enhancement effects and signal amplification functions. Nano-biosensors utilize the unique molecular recognition capabilities of nanomaterials to achieve precise disease detection and are applied in disease diagnosis work (Khan et al., 2024). For example, Zhang et al. (2025) developed a nanosensor based on molybdenum disulfide (MoS_2_), which can detect target microbiota and demonstrated its effectiveness in clinical stool samples, finding that the level of the target microbiota in the feces of CRC patients was higher than in healthy individuals, providing a fast and reliable new method for CRC screening and diagnosis. The LIU team developed a fluorescent sensor based on silver nanoparticles (AgNPs) and vancomycin-modified gold nanoclusters (AuNCs@Van) (Liu Z. et al., 2024). This sensor can distinguish various bacteria, including probiotics, neutral bacteria, and pathogenic bacteria, and can be used for rapid detection of gut microbiota.

Given that specific microbiota, such as *Fusobacterium nucleatum* (*F. nucleatum*), often proliferate in malignant tissues and feces of CRC patients, it has become a key biomarker for early CRC detection. For instance, a study developed a novel nanoprobe (aggregation-induced emission peptide probe (AIE-Pep)) with high selective detection for *F. nucleatum* (detection limit 82.97 colony forming units per milliliter (CFU/mL)), enabling non-invasive screening of CRC (Liu et al., 2025). This probe can precisely locate bacteria in an orthotopic CRC model and significantly differentiate between normal and CRC mouse feces, providing a new strategy for early CRC diagnosis. Besides specific microbiota, certain metabolites can also serve as biomarkers for diagnosing CRC, such as short-chain fatty acids (SCFAs). By detecting changes in SCFA concentrations, early diagnosis and monitoring of various diseases can be achieved. Utilizing this, Platonov and Rumyantseva (2025) used electrospinning to synthesize zinc oxide/metal oxide (ZnO/MOx) nanocomposites. This novel biomarker sensor can monitor human SCFA levels in real-time, helping people better understand their health status, and can serve as a diagnostic scheme for CRC. Furthermore, Chen et al. (2025) combined with near-infrared-II (NIR-II) fluorescence probe imaging technology, used positively charged silver sulfide quantum dots (Ag_2_S QDs) as NIR-II nanoprobes, and efficiently labeled the surface-negatively charged *Escherichia coli* (*E*.*coli*) Nissle 1917 (EcN) via electrostatic interaction. Through NIR-II imaging technology, high spatiotemporal resolution visualization of the gastrointestinal area where the labeled strain is located was achieved, successfully realizing real-time *in vivo* tracking of labeled probiotics, which can be used for early diagnosis of CRC. Therefore, detecting components and biomarkers of gut microbiota through nanotechnology has broad application prospects for diagnosing and treating CRC.

Nanotechnology offers distinct advantages over current “gold standard” methods for CRC screening and diagnosis. In terms of sensitivity, for example, the aforementioned AIE-Pep probe achieves a detection limit of 82.97 CFU/mL for *F. nucleatum*, far lower than the thresholds of traditional culture or conventional polymerase chain reaction (PCR). Similarly, ZnO/MOx nanocomposites exhibit high sensitivity for real-time monitoring of SCFAs. Nanotechnology enables the detection of extremely early and minute biomarker changes, providing potential for early diagnosis and addressing the insufficient sensitivity of methods such as fecal immunochemical test (FIT) in detecting early-stage lesions. Additionally, through surface functionalization modifications—such as vancomycin conjugation or specific peptide probes like AIE-Pep—nanosensors can precisely recognize specific targets (e.g., surface antigens or metabolites of particular bacterial groups), allowing direct detection of microbiota closely associated with CRC pathogenesis and thereby improving diagnostic specificity.

Beyond sensitivity and specificity, nanotechnology provides multidimensional information, enabling quantitative, localized, real-time dynamic monitoring and imaging. For instance, the NIR-II imaging technology developed by Chen et al. permits real-time, high-resolution visual tracking of labeled probiotics within the gastrointestinal tract—a capability unattainable with conventional methods. Moreover, compared to colonoscopy, which requires rigorous bowel preparation and may cause adverse reactions, or traditional microbial detection that relies on days of culturing, nanotechnology-based approaches are more acceptable to patients. Stool sample collection is completely non-invasive, making it suitable for large-scale population screening and convenient application. Therefore, detecting intestinal microbiota components and their biomarkers via nanotechnology holds broad prospects for the diagnosis and treatment of CRC.

### 2.2 Prevention of CRC

The intersection of nanotechnology and microbiome research opens new avenues for CRC prevention. The core lies in using nanomaterials to precisely regulate gut microbiota to intervene in precancerous lesions. Regarding the targeted clearance of pro-carcinogenic pathogens, *F. nucleatum*, as a key pathogenic microorganism, can be specifically cleared by melittin-loaded mesoporous silica coated with colon cancer cell membrane (Mel-SiO_2_@CCM) nanoparticles. This system uses colon cancer cell membrane (CCM) to encapsulate melittin-loaded mesoporous silica to achieve dual targeting, selectively eliminating pathogens without disrupting the ecology of commensal bacteria, markedly preventing pathogen-induced CRC (Wu et al., 2025).

Tryptophan is an important metabolic substrate for gut microorganisms. Metabolism by gut microbiota can produce various indole compounds, which play important roles in preventing CRC. The LI research team designed the engineered EcN with enhanced tryptophan synthesis encapsulated in alginate/gelatin (EcN-TRP@A/G) system, which encapsulates a genetically engineered probiotic, EcN-TRP with enhanced tryptophan synthesis capability (Li et al., 2024). It can rewire the tryptophan metabolic pathway, showing significant preventive and therapeutic effects on mouse colitis, and also provides a new and effective strategy for treating CRC caused by inflammatory bowel disease (IBD). Meanwhile, Zhao Y. et al. (2025) studied DNA nanopatches (DNPs) composed of phage capture strands, modified it onto a specific phage targeting *Streptococcus gallolyticus* (Sg), forming a DNPs@P system. DNPs@P significantly prevented colon tumorigenesis in mouse models by inhibiting the Sg-activated cyclooxygenase-2 (COX-2) pathway and upregulation of *β*-catenin, providing a new perspective for CRC prevention.

While the aforementioned nanotechnological and microbiomic intervention strategies have demonstrated substantial potential for CRC prevention, most current findings are derived from mouse model studies. Their true generalizability, long-term safety, and ultimate preventive efficacy therefore require rigorous validation through more human-relevant models—such as organoids or humanized microbiota mouse models—and future carefully designed early-phase clinical trials. Future research efforts should prioritize bridging the translational gap from animal models to human application, focusing on developing personalized prevention strategies that account for inter-individual variability and can be validated in large-scale clinical trials.

## 3 Nano-delivery systems

Nano-delivery systems are one of the cutting-edge approaches in the field of CRC treatment. Their core value lies in significantly enhancing treatment precision and reducing toxic damage to normal tissues. Compared to traditional delivery systems, nano-delivery systems can optimize oral delivery, address delivery obstacles; achieve precise targeting of tumor tissue; through the design of nanocarriers carrying specific ligands, probiotics and drugs can more accurately accumulate at the tumor site, and can also overcome biological barriers and drug resistance (Chai et al., 2025), providing an efficient, safe, and convenient method for CRC treatment.

### 3.1 Encapsulation and targeted delivery of probiotics

As is well known, the acidic environment of gastric fluid poses the first major challenge for orally delivered probiotics, often leading to significant inactivation. Nanotechnology has achieved notable progress in probiotic delivery systems for CRC therapy. For example, encapsulation techniques using nanomaterials can substantially enhance probiotic survival in the gastrointestinal tract. In this context, Fang and Liu (2024) developed a gelatin-reinforced coating technology to protectively encapsulate probiotics (EcN encapsulated in tannic acid-calcium ion-crosslinked gelatin methacryloyl hydrogel (EcN@TA-Ca^2^+@GelAGE)). They evaluated the survival of coated probiotics in simulated gastric fluid (SGF). After exposing equal amounts (1 × 10^8^CFU) of uncoated and coated live probiotics to SGF for 2 h, all uncoated EcN cells were completely inactivated. In contrast, EcN@TA-Ca^2+^@GelAGE still retained 2.40 Log_10_CFU, demonstrating that the nanomaterial-based coating significantly enhanced protection. The coated probiotics showed approximately 15-fold higher survival in the stomach compared to uncoated EcN. Moreover, even after extended periods of 48 and 96 h, the viable count of EcN@TA-Ca^2+^@GelAGE remained markedly higher than that of the uncoated group, indicating its ability to overcome the harsh gastrointestinal microenvironment. Similarly, Adeel et al. (2023) used nanoliposomes to encapsulate “*Lactobacillus acidophilus*”, achieving *pH*-responsive release through a phospholipid bilayer structure, which also greatly improved probiotic survival and retention. Beyond these approaches, DNA-based microcapsules represent another promising strategy. Such microcapsules can traverse the stomach and duodenum and reach the colon, where the formation and dissociation of A-motif and i-motif structures trigger reversible swelling–shrinking transitions, thereby preserving probiotic viability. Upon disintegration in the colon, the microcapsules release the probiotics to remodel the imbalanced intestinal microbiota (Zhao Z. et al., 2025).

In CRC treatment, probiotics also have defects such as low tolerance and sensitivity to the environment, limiting their effectiveness in the intestine. Ghaznavi et al. (2024) found that coating *Bifidobacterium* with a poly(lactic-co-glycolic acid) nano-coating (PLGA) significantly improved its stability in simulated gastrointestinal environments and enhanced its adhesion ability to intestinal epithelial cells. The involvement of nanotechnology can not only encapsulate probiotics but also achieve targeted release of probiotics. Diep and Schiffman (2024) developed alginate-based nanofibers loaded with probiotics via electrospinning technology for encapsulation and delivery. These nanofibers can protect probiotics from gastric acid and bile salts in simulated gastrointestinal environments and achieve targeted release in the intestines. After immersion in simulated gastric fluid, the probiotics remained viable and encapsulated. Upon transfer to intestinal conditions, the nanofibers released up to 120,000 live probiotic cells per Gram, demonstrating pH-dependent delivery capability. This property significantly enhances the survival and efficacy of probiotics. To improve patient convenience and intestinal acceptance of probiotic therapy, Zhu et al. (2025) designed an orally administered probiotic system, epigallocatechin gallate-polyphenol-armored EcN (ECA@EcN). The ECA coating substantially enhanced intestinal retention and mucosal adhesion of the probiotics. *In vivo* experiments showed that ECA@EcN exhibited prolonged intestinal residence time, with strong fluorescence signals still detectable after 48 h. Its adhesive capacity was 4.3 times higher than that of uncoated EcN. The system maintained structural integrity and bacterial viability under simulated gastric fluid, intestinal fluid, bile salts, and oxidative stress conditions. Furthermore, no significant changes in particle size were observed after incubation in various media, including phosphate-buffered saline (PBS), for 24 h, indicating excellent physical and chemical stability of the coating.

Compared with free probiotics, those coated with nanomaterials exhibit higher survival rates and superior colon-targeting accumulation capacity in the harsh gastrointestinal environment. In models of intestinal diseases such as CRC, encapsulated probiotics are expected to be more efficiently delivered to lesion sites. Through enhanced colonization, local metabolic activity, and potential immunomodulatory effects, they may offer improved therapeutic outcomes. In contrast, free probiotics are susceptible to degradation by gastric acid, bile salts, and other factors, resulting in low viability and insufficient colonization, thereby limiting their therapeutic efficacy. Therefore, nanomaterial-based encapsulation strategies provide a promising solution for enhancing the therapeutic performance of probiotics in diseases like CRC.

### 3.2 Drug delivery

During CRC treatment, complex intestinal barriers and gut microbiota diversity hinder drug delivery efficiency. Factors such as pathogens, mucus, and the epithelial barrier prevent drug-loaded nanoparticles from penetrating, resulting in suboptimal actual therapeutic effects and limited clinical translation. The introduction of nanotechnology can change this phenomenon and greatly improve drug utilization. For example, early on, Wang et al. (2022) designed a biologically chemotactic-guided self-thermophoretic nano-platform for CRC treatment. It incorporated asymmetrically platinum-coated mesoporous silica, allowing the nano-platform to move autonomously in intestinal mucus. Its mucus-penetrating performance increased by 14.6 times, demonstrating good intestinal barrier penetration capability and greatly enhancing drug delivery effectiveness.

During the design of controlled-release systems, a research team designed alginate hydrogel microspheres that integrated *Bifidobacterium* and nano dietary fiber (Qiu et al., 2023). Utilizing microbial fermentation effects, they promoted the targeted release of 5-aminosalicylic acid (5-ASA). The team of LI Yaping from the Chinese Academy of Sciences developed SCXN nanoparticles (Lang et al., 2023), creatively using xylan-stearic acid conjugates self-assembled into nanocarriers to encapsulate capecitabine (Cap). After oral administration, it remains stable in the stomach; after entering the intestine, specific bacteria gradually degrade xylan, releasing the drug steadily and slowly. This design not only reduces the drug absorption rate and increases drug accumulation at the tumor site, but xylan also acts as a prebiotic to promote probiotic growth and short-chain fatty acid production, achieving a synergistic effect of chemotherapy and microbiota modulation.

## 4 Targeted regulation of gut microbiota microenvironment by nanomaterials

Gut microbiota dysbiosis utilizes pathogens like *F. nucleatum* and their carcinogenic metabolites (secondary bile acids, genotoxins) to damage the intestinal mucosal barrier, activating carcinogenic pathways. Traditional interventions face shortcomings such as poor targeting accuracy and low stability. Nanomaterials, by virtue of their unique material properties, can achieve precise targeted control, degradation of carcinogenic metabolites, and improvement of intestinal barrier function (Khalil et al., 2024).

### 4.1 Regulating gut microbiota

Nanomaterials, leveraging their unique physicochemical properties, enable precise control over gut microbiota, mainly manifested in two functions: selective bacteriostasis and ecological environment reconstruction (Liu J. et al., 2024). Regarding selective inhibition of bacteria, nanoparticles synthesized using *Lactobacillus* extracts can release reactive oxygen species (ROS) to destroy the cell membrane and DNA of pathogenic bacteria, showing strong inhibitory effects on pathogens, significantly more effective than traditional antibiotics (such as gentamicin), preventing further tumor development (Suba et al., 2021). Li et al. (2019) combined 4,6-diamino-2-pyrimidinethiol with gold nanoparticles (AuNPs) to design D-Au NPs, which selectively promote the growth of beneficial bacteria and inhibit the proliferation of harmful bacteria, while also protecting intestinal epithelial cells without damaging probiotics in the gut. D-Au NPs can overcome the deficiencies caused by traditional antibiotics, helping to maintain microbiota diversity.

Nanomaterials can not only inhibit the colonization and carcinogenic effects of pathogenic bacteria through oxidative stress but also by disrupting pathogenic biofilms. Lange et al. (2024) designed a graphene oxide (GO)-metal nanoparticle composite (GOM-NPs) and found that GOAg and GOZnO, by releasing metal ions and electron transfer, can not only induce bacteria to produce excessive ROS, causing bacterial oxidative stress and damaging cell membranes, but also the sharp edges of GO directly cut bacterial cell membranes, causing content leakage, thereby inhibiting pathogens. Another research team constructed magnetic mesoporous silica nanoparticles loaded with 6-gingerol (Gin) and performed surface functionalization modification to form P_127_-MLL@Gins nanoparticles (Li B. et al., 2025). Using these nanoparticles significantly increased the abundance of beneficial bacteria (e.g., *Bacillus*) and reduced the proportion of harmful bacteria (e.g., *Bacteroides*), thereby regulating the balance of gut microbiota.

### 4.2 Reducing the impact of gut microbiota metabolites

Carcinogenic metabolites and genotoxins produced by gut microbiota play key roles in the occurrence and development of CRC. These substances can lead to DNA damage, promote inflammation, activate carcinogenic signaling pathways, and other reactions to drive tumor progression (Qu R. et al., 2023). With the continuous application of nanotechnology, carcinogens produced by gut microbiota can be transformed, adsorbed, or cleared, mitigating the impact of metabolites on the pathogenesis and progression of CRC.

*Sulfate-reducing bacteria* (SRB) are the main producers of hydrogen sulfide in the gut. High concentrations of hydrogen sulfide can cause colitis and increase the prevalence of CRC (Dordević et al., 2020). The involvement of nanotechnology provides a new perspective for solving the hydrogen sulfide problem. Porous carbon materials have unique properties, such as large surface area, adjustable porosity, and rich surface chemistry, which can significantly enhance the catalytic oxidation of hydrogen sulfide (H_2_S) at room temperature, promoting the physical adsorption and catalytic oxidation process of hydrogen sulfide (Yang et al., 2022). Wang et al. (2024) developed a copper-exchanged Y zeolite coated with nickel-aluminum layered double oxide (CuY@NiAl-LDO) composite material that achieves catalytic-adsorptive synergistic desulfurization through a core-shell structure. Cu^2+^ in the CuY core layer forms a strong S-M bond with H_2_S (adsorption energy −89.7 kJ/mol), preferentially capturing H_2_S, demonstrating strong adsorption of H_2_S by CuY; the nickel-aluminum layered double oxide (NiAl-LDO) shell acts as a catalyst, catalyzing the hydrolysis of carbonyl sulfide (COS) to generate H_2_S, enhancing H_2_S adsorption efficiency through synergy. Wu et al. (2019) developed an antibiotic release system targeting bacterial toxins based on liposomal nanoreactors, which has good toxin neutralization ability. The principle is that the liposome coating binds to toxins secreted by bacteria, forming stable complexes, thereby neutralizing toxins and significantly reducing the hemolysis rate. Furthermore, studies have used the amphiphilic structure of bile acids to construct microcapsules or nanocapsules for drug delivery and stable encapsulation (Kovacevic et al., 2022). This suggests to us that the interaction mechanism between bile acids and nanomaterials may be used for adsorption design, providing a new research direction for porous nanomaterials (such as activated carbon nanoparticles) to adsorb carcinogenic metabolites produced by bacteria (such as secondary bile acids), blocking their DNA damage to intestinal epithelial cells.

### 4.3 Improving intestinal barrier function

The intestinal barrier, composed of the mucus layer, epithelial cell layer, and immune system, is crucial for preventing infection, inflammation, and food allergies (Wells et al., 2022). The delivery efficiency of both previously mentioned probiotics and drugs is affected by the intestinal barrier, especially the mucus and epithelial barriers hindering the penetration of drug-loaded nanoparticles, leading to unsatisfactory CRC treatment effects. To this end, Hu et al. (2024) developed a novel synergistic delivery system (EcN encapsulated in salicylic acid-functionalized polydopamine-tannic acid nanocomposite (EcN@SA-pBDT-TA)). This nanoparticle can significantly promote the expression of intestinal epithelial tight junction proteins, enhancing intestinal barrier integrity. Moreover, Xu et al. (2023) also designed a selenium nanoparticle-modified EcN (SeM@EcN) nanoparticle, using the membrane derived from EcN as the surface. EcN can strongly adhere to the intestinal mucus layer, prolonging local retention time. SeM scavenges ROS to restore redox balance, protecting intestinal epithelial cells from oxidative damage, and comprehensively repairing intestinal barrier function.

Nanomotors can effectively penetrate the intestinal barrier in CRC treatment, overcoming the physiological obstacles faced by traditional oral drug delivery systems. Wang et al. (2025a) proposed an acoustic-magnetic responsive nanomotor magnetic mesoporous silica nanoparticles (MMSNP) for navigation in the gastrointestinal tract and mucus layer penetration for oral drug delivery. Under ultrasound radiation, it can notably penetrate dense and viscous mucus, thereby improving the bioavailability of loaded drugs. A research team developed a bioactive self-thermophoretic/N_2_ dual-driven nanomotor for CRC treatment (Wang et al., 2025b). This nanomotor can penetrate the intestinal mucus barrier and can reversibly open the paracellular pathway of intestinal epithelial cells, thereby increasing the delivery efficiency of cisplatin by 3.5 times. With its autonomous penetration and reversible regulation of the intestinal barrier ability, it is expected to revolutionize current oral drug delivery strategies.

Therefore, the application of nanomaterials can modulate the intestinal microbiota microenvironment by regulating gut microbiota, reducing metabolite toxicity, and repairing the barrier. This approach enhances the therapeutic efficacy of CRC treatment, significantly improves the richness and diversity of gut microbiota, and helps maintain its balance. A systematic summary of the effects of various nanomaterials on gut microbiota structure and function is presented in Table 1.

**TABLE 1:** Targeted regulation of the gut microbiota microenvironment by nanomaterials.

Type	Nanosystem/Material (Abbr.)	Core design strategy	Target/Key molecule	Main outcomes	Ref.
I Modulating Gut Microbiota	Mammalian Probiotic Extract-NPs	Bacterium-inspired biomimetic ROS release	Pathogen membrane/DNA	Superior antibacterial efficacy vs. gentamicin; blocked tumor progression	Suba et al. (2021)
	D-Au NPs	Dual recognition: surface charge and ligand	Cell wall of harmful bacteria vs. differential metabolic pathways of beneficial bacteria	Selective promotion of probiotics and suppression of harmful bacteria; protected intestinal epithelium	Li et al. (2019)
	GO-Ag/ZnO(GOM-NPs)	Dual-mode of oxidative stress and physical cutting	Pathogenic bacterial biofilm	Disrupted cell membrane, inhibited pathogens	Lange et al. (2024)
	P127-MLL@Gins	Surface functionalization and magnetic targeting	Microbiota structure (↑*Bacillus*, ↓*Bacteroides*)	Increased beneficial bacteria abundance; Decreased harmful bacteria abundance	Li B. et al. (2025)
II Reducing Metabolite Toxicity	CuY@NiAl-LDOCore-Shell Microspheres	Synergistic adsorption-catalysis desulfurization	Hydrogen sulfide and carbonyl sulfide	Enhanced H_2_S adsorption efficiency and clearance	Wang et al. (2024)
	Liposome Nanoreactor	Toxin-triggered membrane fusion release	Bacterial toxins	Significantly reduced hemolysis; strong toxin neutralization	Wu et al. (2019)
	Bile Acid-Nanocapsules	Bile acid amphiphilic structure	Secondary bile acids (carcinogenic metabolites)	Adsorbed carcinogenic metabolites; blocked DNA damage	Kovacevic et al. (2022)
III Repairing Barrier	EcN@SA-pBDT-TA	Upregulation of intestinal epithelial tight junction proteins	Intestinal epithelial tight junction proteins	Enhanced intestinal barrier integrity	Hu et al. (2024)
	SeM@EM	Long-term mucoadhesion and ROS scavenging	Oxidative stress (ROS)	Increased mucus residence time; protected enterocytes from oxidative damage	Xu et al. (2023)
	MMSNP	Ultrasound propulsion and magnetic guidance for mucus penetration	Mucus layer	Increased mucus penetration depth; improved drug bioavailability	Wang et al. (2025a)
	Self-Thermophoretic/N_2_ Dual-Driven Nanomotor	Thermophoresis and gas microbubble reversible opening of paracellular pathway	Paracellular pathway of intestinal epithelium (reversible)	Enhanced cisplatin delivery efficiency; reversible modulation of the intestinal barrier	Wang et al. (2025b)

## 5 Application of nanomaterial-mediated gut microbiota regulation in multi-modal therapy for CRC

Gut microbiota influences CRC treatment response by regulating the immune microenvironment, altering drug metabolic activity, and affecting inflammatory responses. However, general treatment methods (such as surgery, chemotherapy, or immunotherapy) often induce drug resistance due to microbiota imbalance (Wang and Li, 2022). The innovative application of nanomaterials is enabling multi-modal synergistic therapy. Involvement of nanomaterials can compensate for the defects of traditional treatment methods, developing cost-effective, low-side-effect, and highly efficient CRC treatment methods.

### 5.1 Anti-inflammatory therapy

Gut microbiota dysbiosis can lead to the accumulation of toxic metabolites, causing chronic inflammation, which in turn promotes cell transformation into cancer cells, triggering CRC. For example, toxins A/B (TcdA/TcdB) secreted by *Clostridioides difficile* (CD) can promote pro-inflammatory factors such as interleukin-1*β* (IL-1β), causing tumor necrosis factor-*α* (TNF-α) release (Dong et al., 2023); enterotoxigenic *Bacteroides fragilis* (ETBF) produces *Bacteroides fragilis* toxin (BFT), which can simultaneously activate the Wnt/*β*-catenin and nuclear factor kappa-light-chain-enhancer of activated B cells (NF-*κ*B) pathways, promoting cell proliferation and the generation of inflammatory mediators (Sears et al., 2014).

These microbiota-derived toxic metabolites collectively trigger the formation of a chronic inflammatory microenvironment, providing an opportunity for nanotechnology to target and intervene in the microbiota-inflammation-carcinogenesis axis. Targeting the aforementioned mechanisms, nanotechnology offers precise intervention methods. For example, CUI (Cui et al., 2025) constructed an extracellular vesicles-coated UiO-66-NH_2_ metal-organic framework loaded with siRNA (EVs@UiO-66-NH_2_@siRNA) system using *lactobacillus* vesicles to deliver TNF-α small interfering RNA (siRNA). The siRNA can specifically target the key inflammatory factor TNF-α in ulcerative colitis, inhibiting its gene expression, thereby regulating the inflammatory response. Such nanoparticles provide potential targeted therapy for inflammation-induced CRC. The inflammatory microenvironment-responsive microsphere vehicle proposed by Zhao X. et al. (2025) can specifically target colon inflammatory sites, achieving responsive release of MXene and L-arginine. Among them, L-arginine can regulate gut microbiota, improving the balance level of the intestinal microecology; MXene acts as a nano immunoregulatory agent, maintaining immune homeostasis and reducing excessive inflammatory responses, thus enabling the microsphere vehicle to exhibit anti-inflammatory properties. A study combined probiotics, antioxidant nanozymes, and therapeutic drugs to construct a probiotic membrane-modified drug delivery nanocomposite (MPDA@Cur). MPDA@Cur, by scavenging ROS and inducing macrophage M2 polarization, can inhibit the inflammatory response of lipopolysaccharide (LPS)-activated macrophages; it can also regulate the balance of gut microbiota, effectively alleviating the degree of inflammation (Yang et al., 2025). These findings indicate that novel nanocomposites have significant potential in improving the inflammatory microenvironment and implementing comprehensive treatment for CRC.

### 5.2 Enhancing immunotherapy efficacy

Cancer immunotherapy fights tumors by activating the human immune system. Compared with traditional therapies, it has advantages such as low toxicity and good efficacy, but faces challenges such as large patient response differences, immune-related adverse reactions (irAEs), and high treatment costs. Recent studies have found that using nanotechnology to target and regulate gut microbiota can enhance immune cell function and synergize with drugs such as immune checkpoint inhibitors, providing new ideas for improving immunotherapy efficacy (Bergman, 2024).

In several mouse tumor models, Han et al. (2021) made an oral gel from inulin (a common dietary fiber), which can adjust the gut microbial system, increase beneficial bacteria and their produced SCFAs, stimulate systemic memory T cell responses, thereby enhancing the anti-tumor activity of anti-programmed cell death protein 1 (*α*-PD-1), thus strengthening the effectiveness of immunotherapy. This method provides a new strategy for enhancing systemic anti-tumor immunity by *in situ* modulation of gut microbiota. In research on immunotherapy drug delivery systems, Chen et al. (2024) conducted relevant research, developing a bifunctional nano-delivery system DMP-Lac based on *Lactobacillus reuteri* lysate. This system successfully co-delivered lysate and IL-23 A mRNA, achieving a delivery efficiency of 75.56% ± 0.85%. The lysate acts as an immune adjuvant, triggering and regulating immune cells; the delivered IL-23 A mRNA drives changes in downstream immune factors. The two cooperate to achieve efficient anti-cancer immunotherapy effects.

In research surrounding immunotherapy, some teams have also integrated immunotherapy with artificial intelligence (AI). Fan et al. (2024) developed an intelligent tumor therapeutic micro-robot (VA-SAM@BTO) based on Veillonella atypica (VA). This system can synchronously catalyze multiple redox reactions, inducing immunogenic death (ICD) of tumors. Moreover, VA-SAM@BTO has the function of remodeling the immune microenvironment, promoting immune cell activation, achieving the synergistic implementation of catalytic therapy and immunotherapy, greatly enhancing the anti-tumor effect, and providing a new targeted treatment strategy for CRC.

### 5.3 Radiosensitization/chemosensitization and reduction of toxic side effects

There are many limitations in CRC chemotherapy (Yixia et al., 2021) and radiotherapy (Long et al., 2024), such as direct damage to intestinal epithelial cells, causing changes in the local microenvironment, inducing microbiota dysbiosis (e.g., decreased *Lactobacillus* content, increased *Proteobacteria*), and toxic side effects caused by chemotherapy drugs. Nanocarriers achieve synergistic therapy by integrating chemotherapy drugs and microbiota modulation components. As one of the ways to surpass traditional cancer treatment technologies, nanomedicine, by modulating gut microbiota, can greatly enhance the overall therapeutic effect of cancer chemoradiotherapy.

Recent studies have found that CRC patients undergoing Cap treatment experience significant changes in the composition of gut microbes (Hillege et al., 2025), and during chemotherapy, there are phenomena such as poor drug delivery system efficiency and significant off-target toxicity. Regulating microbiota is crucial in CRC treatment. Sun et al. (2025) for the first time integrated a chemotherapy drug (5-fluorouracil, 5-FU), a prebiotic (Sxy), and a probiotic active ingredient (DPPE) into the same nanocarrier, synthesizing nano-liposomes FLSK. Among them, Sxy can regulate microbiota, reduce intestinal inflammation and mucosal damage, and reduce various side effects caused by chemotherapy. Ma et al. (2024) used nanotechnology and immunotherapy for synergistic treatment, developing a multifunctional oral dextran-aspirin nanomedicine that can target scavenge ROS and release salicylic acid. It uses dextran prebiotic to regulate gut microbiota, stimulating the growth of anti-cancer microbiota (*Lactobacillus* increased 6.6 times, *Akkermansia* increased to 103 times the original), synergistically treating CRC. Chemotherapy drugs can change the gut microbiota by affecting intestinal barrier integrity, thereby reducing the therapeutic effectiveness of cancer chemotherapy. Lang et al. (2023) used prebiotic xylan-stearic acid conjugate (SCXN) nanoparticles to deliver Cap. SCXN can enhance anti-tumor immunity by modulating microbiota (promoting probiotic growth, producing SCFAs). Experiments confirmed that compared to free Cap, SCXN significantly increased the tumor inhibition rate (from 5.29% to 71.78%) and extended the median survival time of tumor-bearing mice. This prebiotic-based nanoparticle, combining gut microbiota modulation with chemotherapy, presents a promising CRC treatment method.

Radiotherapy is a primary method for treating solid tumors but may be associated with unacceptable levels of off-target tissue toxicity, affecting treatment efficacy and patient quality of life (Al-Qadami et al., 2019). Gut microbiota is important for the immune system. Radiotherapy can damage the intestinal mucosa, disrupt gut microbiota balance, and affect the immune system. Therefore, during radiotherapy, drug delivery systems are needed to re-establish gut microbiota homeostasis and achieve optimal chemoradiotherapy effects with minimal side effects. Shi et al. (2022) designed a thiol-crosslinked bacterial cellulose hydrogel (SulBC gel) and loaded the anticancer drug cisplatin (CDDP) into the SulBC gel to form CDDP@SulBC gel. This gel can increase X-ray-induced DNA double-strand breaks, induce G2/M phase arrest in tumor cells, significantly increase the sensitivity of intestinal tumor cells to radiotherapy, and re-establish gut microbiota balance, helping to alleviate radiation-induced colitis. Similarly, to reduce radiation-induced intestinal injury from radiotherapy, He et al. (2024) developed a nanoparticle platform composed of mesoporous silica (MS), ZIF-8 (metal-organic framework material), and SI, named SI@ZIF-8@MS NP. In mouse experiments, it was found that treatment with SI@ZIF-8@MS could alleviate radiation-induced gut microbiota dysbiosis and restore radiation-induced changes in bacterial composition. Currently, combining nanotechnology with chemoradiotherapy to improve the therapeutic effect of CRC by regulating gut microbiota has shown significant results and is expected to become a clinical strategy.

These studies collectively confirm that nanomaterials, through precise regulation of the “microbiota-immune-chemotherapy/radiotherapy” interaction network, can not only overcome the toxic side effects of traditional chemoradiotherapy but also improve therapeutic efficacy through microbiota remodeling, providing innovative solutions for multi-modal treatment of CRC. The field has now shifted from mere radio/chemo-sensitization to developing synergistic strategies of “targeted delivery-microbiota regulation-immune activation”, possessing significant clinical translation potential.

### 5.4 Nanotechnology overcomes drug resistance and tumor metastasis

Like other cancers, CRC also faces significant challenges of chemotherapy resistance and tumor metastasis. In addition to acquired resistance induced by long-term medication, certain genomic instabilities in CRC also drive the occurrence of intrinsic resistance mutations. For example, *F. nucleatum* may promote tumor growth and lead to chemotherapy resistance (Chen et al., 2021). Therefore, regulating gut microbiota has become a new strategy for treating CRC resistance. In the early stages of CRC, short-chain fatty acid butyrate can be used to downregulate the expression of relevant outer membrane proteins, inhibiting the growth, enrichment, and adhesion of *F. nucleatum* in CRC tissues. Sodium butyrate (NaBu) encapsulated in liposomes can reduce the colonization and invasion of *F. nucleatum*, thereby alleviating its induced chemotherapy resistance (Chen et al., 2023). Drug tolerance exists not only in chemotherapy but also in immunotherapy. CD8 T cell dysfunction may be a key factor leading to immunotherapy resistance. A research team combined nanotechnology and probiotic therapy to develop a selenium nanovesicles loaded with nano-emulsion-encapsulated interleukin-32 and EcN (SeNVs@NE-IL32-EcN) complex. This complex not only enhanced the proliferation and activity of CD8 T cells but also overcame the drug resistance of CRC immunotherapy (Li S. et al., 2025).

As mentioned before, *F. nucleatum* is one of the main pathogens of CRC. It not only leads to poor treatment effects but may also be an important cause of tumor metastasis. Therefore, regulating intratumoral *F. nucleatum* to affect the tumor microenvironment (TME) can enhance the therapeutic effect of CRC and inhibit lung metastasis. Qu X. et al. (2023) designed an ultrasound-stimulated responsive nano-platform (Au@BSA-CuPpIX), which can clear *F. nucleatum* to inhibit tumor growth and metastasis. The nanoparticle Mel-SiO_2_@CCM, which has CRC preventive effects, also has such ability (Wu et al., 2025). Mel in this nanoparticle can destroy bacterial cell membranes, clearing intratumoral *F. nucleatum*; homologous targeting promotes the internalization of nanoparticles into tumor cells, inducing apoptosis. It achieves targeted clearance of pro-cancer bacteria and immune activation, eradicating carcinogenic microbiota-related metastases, thereby reducing cancer metastasis risk. Besides inhibiting tumor metastasis by clearing pathogens, some teams have used gene editing technology to inhibit tumor development (Ding et al., 2024). They used CRISPR-Cas9 edited tumor cell membrane-modified nanoparticles to co-deliver cyclin-dependent kinase (CDK) inhibitors and programmed death-ligand 1 (PD-L1) antibodies; they can homologously target liver metastases, induce immunogenic death, reverse the immunosuppressive microenvironment, inhibit the secretion of immunosuppressive cytokines (such as interleukin-10 (IL-10), transforming growth factor-*β* (TGF-*β*)), and reduce the suppression of the immune system. This can obviously reduce the risk of colon cancer liver metastasis and provide a better prognosis for patients.

## 6 Limitations of nanotechnology applications

When nanomaterials are applied in the biomedical field, their biocompatibility becomes a critical consideration (Wei et al., 2024). In recent decades, the field of nanobiotechnology has achieved significant breakthroughs. Naturally derived and chemically synthesized nanobiomaterials, typically ranging from 1 to 100 nanometers in size, have been extensively utilized across various domains, particularly in medicine. However, this advancement has not been without challenges, as several core bottlenecks remain, such as reproducibility, biocompatibility, biosafety, long-term toxicity, manufacturability, and regulatory compatibility. The precise control required during nanomaterial synthesis amplifies the risk of batch-to-batch variations; minor fluctuations in temperature, solvent purity, or purification parameters may alter the final product, leading to markedly different therapeutic effects in vitro cell experiments and *in vivo* animal models, thereby significantly compromising treatment consistency. Furthermore, the therapeutic performance of nanomaterials in complex biological environments—such as the gastrointestinal *pH* gradients, bile salts, digestive enzymes, mucus layers, and dynamic microbiota—exhibits nonlinear and highly sensitive responses, which affects their reproducibility in therapeutic applications.

To develop effective and safe nanomedicines, a thorough understanding of the physical properties of nanoparticles—including composition, morphology, structural features, chemical properties, surface charge, and aggregation behavior—is essential (Venturini et al., 2025). These characteristics critically influence the biocompatibility of nanoparticles and may limit their application scope. Current applications of nanomaterials also face potential acute or long-term toxicity concerns, which could compromise cellular integrity, trigger inflammatory responses, or induce DNA damage, thereby constraining their *in vivo* use. Safety issues related to nanomaterials span multiple dimensions, encompassing intrinsic material toxicity, bioaccumulation, and potential impacts on the immune system (Pradhan et al., 2021).

Moreover, scaling up the manufacturing of nanomaterials and nanodevices to industrial levels presents considerable complexity. Transitioning from laboratory-scale synthesis to mass production remains challenging and requires careful process optimization (Beura et al., 2024). For clinical translation of nanomaterials in CRC therapy, large-scale manufacturing remains a major obstacle: the intricate synthesis processes of nanomedicines often do not align with industrial-scale production requirements, which hinders widespread clinical adoption. A core priority in scaling up production is ensuring the reliability and consistent batch-to-batch reproducibility of nanomedicines. Advancing nanotechnology for CRC treatment therefore involves multifaceted challenges that require coordinated efforts among researchers, industry stakeholders, and regulatory authorities. By addressing these issues collectively, nanotechnology can progress in a safe, sustainable, and responsible manner, ultimately delivering greater benefits to society.

## 7 Conclusion

**FIGURE 1:**
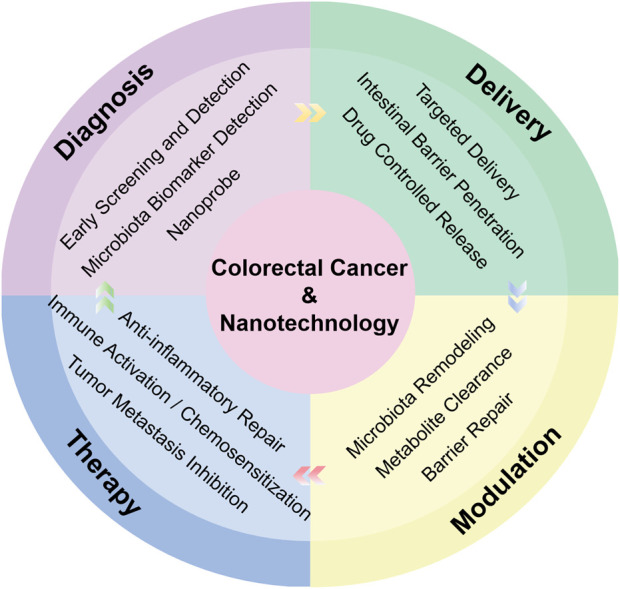
Schematic overview of nanotechnology for the diagnosis, delivery, modulation and therapy in CRC via gut microbiota modulation.

This review comprehensively explores the application prospects of nanotechnology in CRC treatment. While current primary approaches for CRC—surgery, chemotherapy, and radiotherapy—remain the mainstay of clinical management, their long-term use is often constrained by toxic side effects, drug resistance, and recurrence risks. Over the past two decades, targeted therapy and immunotherapy have significantly improved patient prognosis; however, challenges such as acquired resistance and low response rates persist. Recent studies highlight the pivotal role of gut microbiota modulation in CRC prevention, treatment efficacy, and the regulation of toxicity associated with chemo- and radiotherapy. This article focuses on the breakthroughs achieved in nanomedicine, critically reviewing advances in nanotechnology for modulating intestinal flora, probiotic delivery, and drug-carrier systems. It also elucidates novel strategies encompassing nano-enhanced immunotherapy, chemoradiosensitization, early precise diagnosis, and intestinal barrier repair (Figure 1).

The therapeutic outcomes of nanotechnology combined with other modalities have evolved from a simple additive effect to a multidimensional synergistic and systemic treatment paradigm. Its clinical value lies not only in enhanced efficacy but also in fundamentally overcoming the inherent limitations of conventional therapies. Nano-platforms are not merely passive “delivery vehicles”; rather, they function as intelligent regulatory hubs that achieve spatiotemporally precise control, microenvironment reprogramming, and coordinated modulation of signaling pathways, thereby substantially improving the effectiveness of radiotherapy, chemotherapy, immunotherapy, and targeted treatment.

Innovative applications of nanomedicine based on gut microbiota regulation have significantly enhanced the diagnostic accuracy and treatment efficiency of CRC, prompting revolutionary breakthroughs in the application of nanotechnology in CRC diagnosis and treatment. Although nanotechnology has great potential in regulating gut microbiota and controlling the occurrence and development of CRC, challenges such as biocompatibility, targeting, complexity of the intestinal microenvironment, immune response, clinical translation, and drug resistance still need to be addressed. Future research should further focus on the design and optimization of nanomaterials and their interaction mechanisms with gut microbiota, thereby providing more effective and safer measures for the treatment of CRC. Moreover, it is necessary to implement multi-omics integration, explore new treatment methods, and simultaneously promote interdisciplinary collaboration to jointly achieve precision and efficiency in the prevention and treatment of CRC.
